# DeepSeek-R1 and GPT-4 are comparable in a complex diagnostic challenge: a historical control study

**DOI:** 10.1097/JS9.0000000000002386

**Published:** 2025-04-03

**Authors:** Lining Chan, Xinjie Xu, Kaiyang Lv

**Affiliations:** a Department of Plastic Surgery, Xinhua Hospital, Shanghai Jiao Tong University School of Medicine, Shanghai, People’s Republic of China

**Keywords:** artificial intelligence (AI) in medicine, clinical decision support, deepSeek-R1, diagnostic accuracy, large language models (LLMs), medical diagnosis

## Abstract

**Background:**

Large language models (LLMs) have demonstrated potential in medical diagnostics, but their accuracy in complex cases remains a subject of investigation. DeepSeek-R1, an open-source model with advanced reasoning capabilities, has gained global attention. This study evaluates the diagnostic performance of DeepSeek-R1 compared to GPT-4 in complex clinical cases.

**Materials and methods:**

A historical control study was conducted using 100 clinicopathologic cases from the *New England Journal of Medicine* (NEJM), published between 18 August 2022, and 30 January 2025. Each case was processed using DeepSeek-R1 with a structured diagnostic prompt. The model’s performance was assessed based on final diagnosis accuracy, differential diagnosis inclusion rate, ranking of correct diagnoses, and differential quality scores. Results were statistically compared to previously published GPT-4 performance data using chi-square, Mann-Whitney U, and t-tests.

**Results:**

DeepSeek-R1 correctly matched the final diagnosis in 35% of cases (35/100), which was comparable to GPT-4’s accuracy (39%; *P* = 0.634). However, DeepSeek-R1 included the correct diagnosis in its differential list in 48% of cases, significantly lower than GPT-4 (64%; *P* = 0.036). DeepSeek-R1 generated longer differential diagnoses (11.9 ± 2.0 vs. 9.0 ± 1.4; *P* = 0.000004) but maintained a similar mean rank for correct diagnoses (1.8 ± 2.2 vs. 2.5 ± 2.5; *P* = 0.288566) and equivalent differential quality scores (4.2 ± 0.10 vs. 4.2 ± 1.3; *P* = 0.099667).

**Conclusion:**

DeepSeek-R1 exhibits diagnostic accuracy comparable to GPT-4 while generating more diverse differential diagnoses. Its open-source nature and innovative reasoning strategies may enhance medical AI applications. Future studies should explore real-world clinical integration and refinement of differential diagnosis prioritization.

Diagnostic applications of large language models (LLMs) in the medical field have garnered significant attention. LLMs’ diagnostic performance in complex cases reflects their comprehensive clinical diagnostic capabilities. Kanjee *et al* have found that Generative Pre-trained Transformer 4 (GPT-4) showed great potential in such settings^[^1^]^. The emergence of new LLMs may indicate an improvement in the diagnostic capabilities for complex cases. DeepSeek-R1, as an open-source LLM with reasoning capabilities has gained great attention globally, and its accuracy in complex diagnostic challenges still needs to be evaluated.

**HIGHLIGHTS**

- DeepSeek-R1 and GPT-4 showed comparable diagnostic accuracy in complex cases.
- DeepSeek-R1 generated more diverse differential diagnoses than GPT-4.
- The inclusion rate of correct diagnoses in differentials was lower in DeepSeek-R1 (48% vs. 64%, *P* = 0.036).
- Both models achieved similar differential quality scores (*P* = 0.099667).
- Findings suggest DeepSeek-R1’s potential in AI-assisted medical diagnosis.

## Method

**Figure 1.**
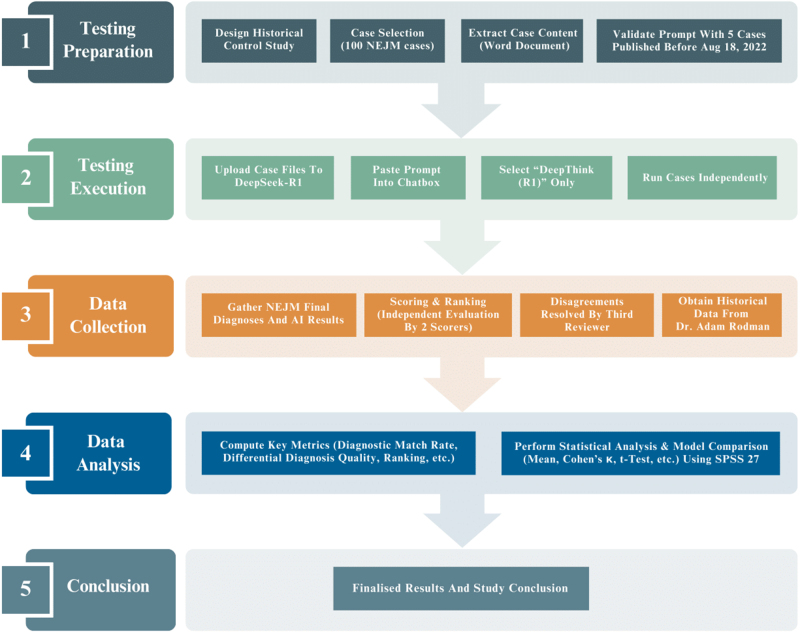
DeepSeek-R1 was tested on complex diagnostic challenge as GPT-4 had been performed previously^[^1^]^. The results of DeepSeek-R1 were statistical compared with those of GPT-4ʹ.

We designed a historical control study to test the ability and accuracy of DeepSeek-R1 to diagnose complicated medical cases, as described in Fig. 1. A test dataset of 100 cases from the *New England Journal of Medicine (NEJM)* clinicopathologic conferences, published from 18 August 2022, to 30 January 2025, was collected. The content of each case, including tables and descriptive captions for images, up to but not including the discussant’s initial response and the differential diagnosis discussion, was saved as a separate Microsoft Word document. We used a prompt (Supplement 1, http://links.lww.com/JS9/E49) similar to that in Kanjee’s study and tested its correct performance with five cases published before August 18, 2022^[^1^]^.

In the testing stage, the organized case file was uploaded to DeepSeek-R1’s official website, and the prompt was pasted into the chat box. The option of “DeepThink (R1)” was chosen while the option of “Internet Search” was not chosen before searching for the results. Each case was run independently. We collected the final diagnosis from the *NEJM* clinicopathologic conferences, the final diagnosis provided by DeepSeek-R1, and its differential diagnoses. Percentage of cases where the artificial intelligence (AI) final diagnosis matched the case final diagnosis, percentage of final diagnosis included in the differential diagnosis, length of differential diagnoses, rank of the correct diagnosis in its differential, and differential quality score were calculated as previous described^[^1^]^. All cases were independently assessed by Lining Chan and Xinjie Xu, and any disagreements were resolved by Kaiyang Lv. The original data were generously provided by Dr. Adam Rodman for statistical comparison^[^1^]^. SPSS 27 (IBM) was used for descriptive statistics, interrater reliability (assessed with Cohen’s κ), and for comparing differences between current and historical data using the chi-square test, Mann-Whitney U test, and t test when necessary.

## Results

**Figure 2.**
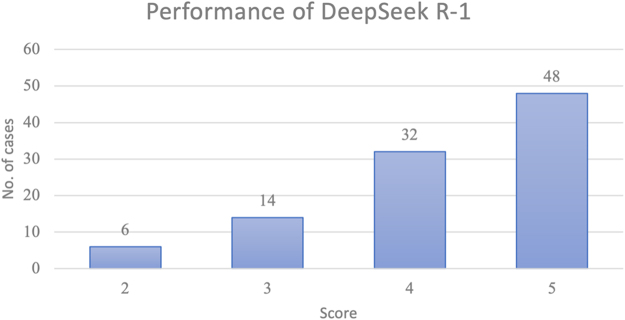
Histogram of DeepSeek-R1’s performance. Performance scale scores^[^1^]^: 5 = the actual diagnosis was suggested in the differential; 4 = the suggestions included something very close, but not exact; 3 = the suggestions included something closely related that might have been helpful; 2 = the suggestions included something related, but unlikely to be helpful; 0 = no suggestions close to the target diagnosis. (The scale does not contain a score of 1.)

All cases (*n* = 100) were able to correctly generate the predefined outcomes. The two primary scorers agreed on 72% of the scores (72/100; κ = 0.565 [moderate agreement]). The primary diagnosis provided by DeepSeek-R1 matched the final diagnosis in 35% (35/100) of cases, which is not significantly different from that of GPT-4 (39% (27/70)). In 2 cases, the final diagnosis included two diagnoses, whereas DeepSeek-R1 provided only one diagnosis, and these conditions were not counted as correct diagnoses. The percentage of final diagnoses included in the differential by DeepSeek-R1 was lower than that of GPT-4 (DeepSeek-R1 vs. GPT-4: 48% vs. 64%, *P* = 0.036). The length of differential diagnoses of DeepSeek-R1 is longer than that of GPT-4 (DeepSeek-R1 *vs.* GPT-4: 11.9 ± 2.0 *vs.* 9.0 ± 1.4, *P* = 0.000004). In cases where the AI model included the correct diagnosis in its differential, the mean rank of the diagnosis did not differ between the two LLMs (DeepSeek-R1 *vs.* GPT-4: 1.8 ± 2.2 *vs.* 2.5 ± 2.5, *P* = 0.288566) (Table 1). Approximately half of the cases received the highest differential quality score (Fig. 2), and the median differential quality score also showed no difference between two LLMs (DeepSeek-R1 *vs.* GPT-4: 4.2 ± 0.10 vs. 4.2 ± 1.3, *P* = 0.099667) (Table 1).

**Table 1:** Performance comparison on complex diagnostic challenge between DeepSeek-R1 and GPT-4

Performance metric	DeepSeek-R1 (n = 100)	GPT-4 (n = 70)	*P* value
Percentage of cases where the AI final diagnosis matched the case final diagnosis (%)	35 (35/100)	39 (27/70)	0.634
Percentage of final diagnosis included in differential (%)	48 (48/100)	64 (45/70)	0.036
Length of differential diagnoses	11.9 ± 2.0	9.0 ± 1.4	0.000004
Rank of the correct diagnosis in its differential	1.8 ± 2.2	2.5 ± 2.5	0.288566
Differential quality score	4.2 ± 0.10	4.2 ± 1.3	0.099667

AI, artificial intelligence; GPT4, generative pre-trained transformer 4.

## Discussion

This study systematically compared the performances of DeepSeek-R1 and GPT-4 in complex medical diagnostic tasks. The results showed no significant difference between DeepSeek-R1 and GPT-4 in terms of final diagnostic accuracy (35% vs. 39%, *P* = 0.634), and the average rank of correct diagnoses in differential diagnoses was also similar (1.8 ± 2.2 vs. 2.5 ± 2.5, *P* = 0.288566), indicating that both models have comparable core diagnostic capabilities. Furthermore, there was no significant difference in the quality scores of differential diagnoses (4.2 ± 0.10 vs. 4.2 ± 1.3, *P* = 0.099667), further supporting the comparability of their diagnostic quality. However, the proportion of differential diagnoses including the final diagnosis was lower for DeepSeek-R1 than for GPT-4 (48% vs. 64%, *P* = 0.036). This may be because DeepSeek-R1 generated more differential diagnoses (11.9 ± 2.0 vs. 9.0 ± 1.4, *P* = 0.000004), reflecting its advantage in generating diverse differential diagnoses. Nonetheless, considering that there was no difference in the core diagnostic capabilities between the two LLMs, the diversity in differential diagnoses generated by DeepSeek-R1 does not appear to have resulted in an increased diagnostic rate. Overall, DeepSeek-R1 demonstrated greater diversity in differential diagnoses while maintaining accuracy and quality in core diagnostics comparable to those of GPT-4, providing strong support for its application in the medical field (Table 1). We attributed these results to DeepSeek-R1’s innovative techniques, including mixture-of-experts and multi-head latent attention, which boost the model’s efficiency with significantly lower training costs^[^2,3^]^.

The DeepSeek-R1 model claims that its training data is up to July 2024 and does not include content from medical journals like NEJM that require subscriptions or paid access. Additionally, our case diagnosis process eliminated the possibility of network functionalities and self-learning. We believe that the research results reflect the inherent capabilities of the model, and there is no possibility of “cheating” through pre-training on the evaluated data or internet searches.

This study has limitations. First, although control study data were obtained for statistical analysis, the historical control design may have introduced errors due to selection bias and confounding factors. Second, differential quality scores were assessed manually, which may have introduced subjectivity.

DeepSeek-R1 offers open-source algorithmic innovation with “chain of thought” reasoning, overcoming the “black box” disadvantage often seen in closed-source models such as GPT-4^[1,^^3^,^4^^]^. This presents a new development strategy for AI in medicine, enhancing the efficiency of medical diagnosis and education through algorithmic innovation and open-source strategies^[^5^]^ based on traditional LLMs. We remain optimistic about AI’s deeper integration into the medical field.

## Data Availability

The datasets generated and analyzed during this study will be publicly available upon publication. A detailed dataset is provided in the supplementary file (Supplement 2.docx) and can be accessed by contacting the author via email.
